# Platelet-derived growth factor-C promotes human melanoma aggressiveness through activation of neuropilin-1

**DOI:** 10.18632/oncotarget.18706

**Published:** 2017-06-27

**Authors:** Federica Ruffini, Lauretta Levati, Grazia Graziani, Simona Caporali, Maria Grazia Atzori, Stefania D’Atri, Pedro M. Lacal

**Affiliations:** ^1^ Laboratory of Molecular Oncology, “Istituto Dermopatico dell’Immacolata”-IRCCS, Rome, Italy; ^2^ Department of Systems Medicine, University of Rome Tor Vergata, Rome, Italy

**Keywords:** PDGF-C, neuropilin-1, melanoma, metastatic phenotype, Snail

## Abstract

Despite recent progress in advanced melanoma therapy, identification of signalling pathways involved in melanoma switch from proliferative to invasive states is still crucial to uncover new therapeutic targets for improving the outcome of metastatic disease. Neuropilin-1 (NRP-1), a co-receptor for vascular endothelial growth factor-A (VEGF-A) tyrosine kinase receptors (VEGFRs), has been suggested to play a relevant role in melanoma progression. NRP-1 can be activated by VEGF-A also in the absence of VEGFRs, triggering specific signal transduction pathways (e.g. p130Cas phosphorylation). Since melanoma cells co-expressing high levels of NRP-1 and platelet derived growth factor-C (PDGF-C) show a highly invasive behaviour and PDGF-C shares homology with VEGF-A, in this study we have investigated whether PDGF-C directly interacts with NRP-1 and promotes melanoma aggressiveness. Results demonstrate that PDGF-C specifically binds *in vitro* to NRP-1. In melanoma cells expressing NRP-1 but lacking PDGFRα, PDGF-C stimulates extra-cellular matrix (ECM) invasion and induces p130Cas phosphorylation. Blockade of PDGF-C function by neutralizing antibodies or reduction of its secretion by specific siRNA inhibit ECM invasion and vasculogenic mimicry. Moreover, PDGF-C silencing significantly down-modulates the expression of Snail, a transcription factor involved in tumour invasiveness that is highly expressed in NRP-1 positive melanoma cells. In conclusion, our results demonstrate for the first time a direct activation of NRP-1 by PDGF-C and strongly suggest that autocrine and/or paracrine stimulation of NRP-1 by PDGF-C might contribute to the acquisition of a metastatic phenotype by melanoma cells.

## INTRODUCTION

Cutaneous melanoma is an extremely aggressive skin cancer with a highly metastatic potential. Indeed, melanoma spreading to distant organs is the primary cause of skin cancer-related deaths. Despite advances in the therapy of the metastatic disease with the approval of immune checkpoints inhibitors (i.e. ipilimumab, pembrolizumab and nivolumab) and, for BRAF mutated melanomas, of BRAF and MEK inhibitors (vemurafenib, dabrafenib, trametinib, cobimetinib), melanoma patients diagnosed at advanced stages have low expectancy of life [1].

The molecular mechanisms associated with the acquisition of a metastatic phenotype by melanoma cells are not very well defined and, therefore, the identification of signalling pathways involved in the metastatic switch is of primary importance. Melanoma progression is greatly favoured by angiogenesis and appears to be associated with an increase of vascular endothelial growth factor-A (VEGF-A) expression by tumour cells [2]. VEGF-A interacts with two high-affinity transmembrane tyrosine kinase receptors (VEGFR-1 and VEGFR-2) [3], but it also binds to neuropilin-1 (NRP-1), a transmembrane polypeptide that, acting as co-receptor, amplifies the signal transmitted by VEGF-A through VEGFR-2 [4]. In the absence of VEGFR-2, NRP-1 can still be activated by VEGF-A. Expression of NRP-1 in melanoma cells correlates with tumour cell invasive behaviour and formation of tube-like structures resembling vascular networks (vasculogenic mimicry). These effects derive from up-regulation of VEGF-A, matrix metalloprotease (MMP-2, MMP-9) secretion [5] and activation of specific αv integrins [6]. High NRP-1 expression is also involved in tumour resistance to VEGF-A blocking therapies [7], suggesting that inhibition of NRP-1 may be required to prevent compensatory escape mechanisms by tumour cells.

Most of the approaches targeting NRP-1 investigated so far are based on the blockade of its ability to bind VEGF-A or to interact with VEGFR-2. However, an anti-NRP-1 humanized antibody that hampers VEGF-A binding to NRP-1, even though well-tolerated as single agent, showed modest clinical activity [8]. Interestingly, NRP-1, acting as co-receptor, can bind to and be stimulated by growth factors different from VEGF-A, such as placenta growth factor, tumour growth factor and platelet derived growth factor-B (PDGF-B) (reviewed in reference 4). Moreover, we previously reported that human melanoma cells, which co-express high PDGF-C and NRP-1 levels, show a marked invasive behaviour suggesting a relevant role for both molecules in the promotion of melanoma aggressiveness [9].

PDGF-C is a growth factor that, unlike the more extensively studied PDGF family members PDGF-A and PDGF-B, contains a CUB (complement C1r/C1s, Uegf, Bmp1) domain preceding the growth factor core region. CUB domain must be proteolytically removed to allow PDGF-C binding to its receptor [10] (PDGFR), which is a dimeric protein composed of two different chains, α and β. PDGF-C homodimer has a high affinity for the PDGFRα chain, and binds preferentially to the αα homodimeric form of the receptor. However, when PDGFRα and PDGFRβ are co-expressed in the same cell, PDGF-C can also bind to the PDGFRαβ heterodimer [11].

Interaction of PDGF-C with PDGFRα promotes tumour growth by several mechanisms, acting as: survival and mitogenic factor for tumour cells, mitogenic and chemoattractant factor for cancer-associated fibroblasts and inducer of tumour angiogenesis in a VEGF-A-independent way [12, 13]. Indeed, PDGF-C induces angiogenesis due to its ability to recruit endothelial cells, pericytes and smooth muscle cells. It also stimulates fibroblasts and macrophages, which are a source of angiogenic factors and proteases required for pathological angiogenesis [12]. Moreover, since PDGF-C protects macrophages from apoptosis, cancer cells secreting this cytokine might favour the recruitment of tumour-associated macrophages [14].

Based on PDGF-C higher structural homology to VEGF-A than to other PDGF family members and on VEGF-A ability to stimulate NRP-1 [15], we have investigated whether PDGF-C activates NRP-1 and cooperates with it in melanoma progression, likely favouring the metastatic switch. Results indicated that PDGF-C directly interacts with NRP-1 *in vitro* and stimulates ECM invasion and vasculogenic mimicry in human melanoma cells expressing NRP-1 but lacking other VEGFRs and PDGFRs. Our data indicate that the highly aggressive phenotype displayed by NRP-1 proficient melanoma cells is sustained, at least in part, by a PDGF-C/NRP-1 autocrine loop.

## RESULTS

### A PDGF-C-directed autocrine loop promotes migration of NRP-1 expressing melanoma cells

**Figure 1:**
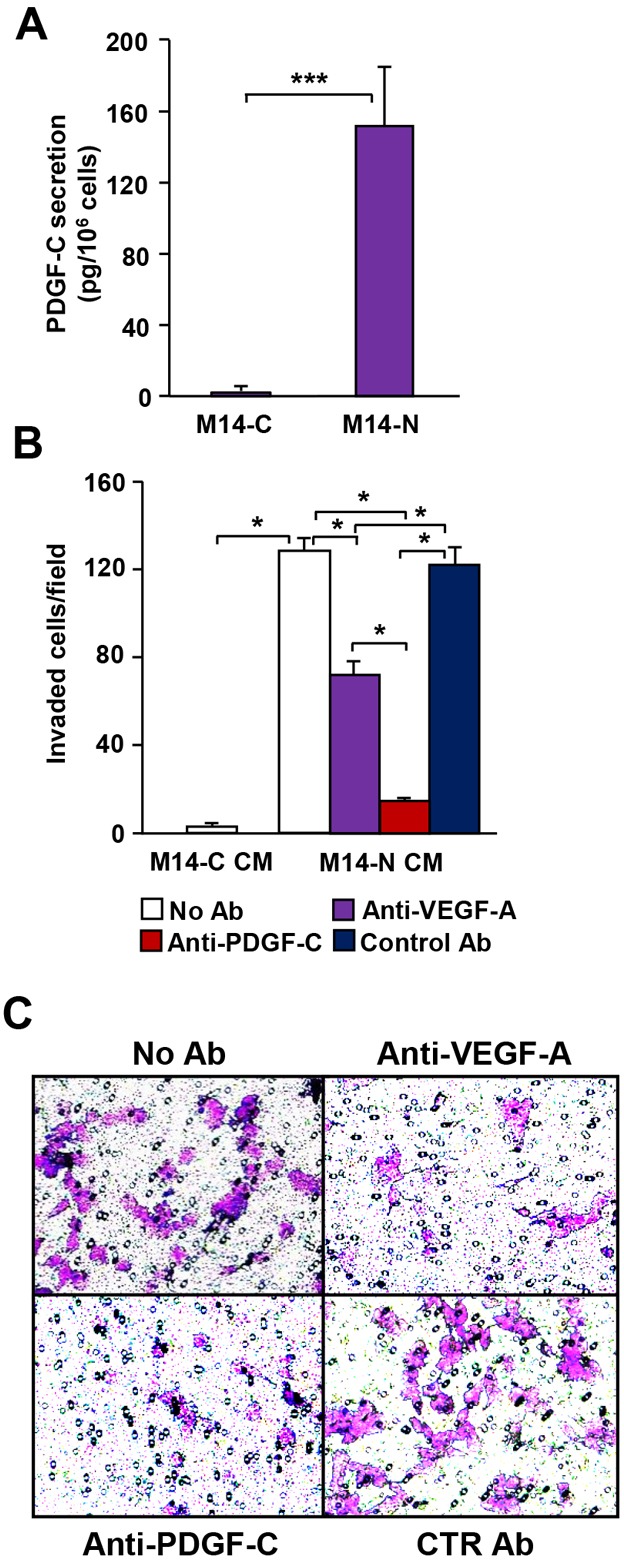
An autocrine loop involving PDGF-C promotes invasiveness of M14-N melanoma cells **(A)** Analysis of PDGF-C secretion by M14-N and M14-C cells was measured in cell culture supernatants by ELISA. Each value is the mean of three independent determinations (± SD). Student’s *t*-test: p<0.001 (***). **(B)** Matrigel invasion by M14-N cells in response to conditioned medium (CM) obtained from M14-N cells or M14-C cells. Invasion of melanoma cells, untreated (No Ab) or in the presence of blocking antibodies specific for VEGF-A or PDGF-C or with IgG control antibody, was analysed using Boyden chambers equipped with matrigel-coated filters and including CM in the lower compartment. Each value represents the mean of three independent experiments (± SD). ANOVA followed by Bonferroni’s post-hoc test: p<0.05 (*). **(C)** Selected representative microscopic fields (x100 magnification) are shown that correspond to M14-N cells stimulated with M14-N conditioned medium in the absence of antibodies (No Ab), and in the presence of 5 μg/ml goat polyclonal anti-VEGF-A, anti-PDGF-C or IgG control antibodies (CTR Ab).

Studies were performed using two previously described human melanoma cell clones (M14-C and M14-N), which lack VEGFR-1 and VEGFR-2, but differ in NRP1 expression and invasiveness [5, 6]. Compared to NRP-1 negative M14-C cells, NRP-1 positive M14-N cells have an extremely aggressive and invasive phenotype [5, 6, 16]. Analysis of PDGF-C in culture medium revealed that M14-N cells secreted PDGF-C at 600-fold higher levels than M14-C cells (Figure 1A). Conditioned medium collected from M14-N cells promoted their invasion through ECM and this effect was strongly reduced by an anti-PDGF-C antibody, to a higher extent as compared with a VEGF-A neutralizing antibody (Figure 1B and 1C), which is also released at high levels by M14-N cells [5]. By contrast, supernatants from the less aggressive M14-C cell clone did not stimulate M14-N cell invasiveness in the same experimental conditions (Figure 1B). Moreover, M14-N conditioned medium did not induce ECM invasion by NRP-1 negative M14-C cells (Supplementary Figure 1).

### PDGF-C directly interacts with NRP-1 *in vitro* and stimulates NRP-1 signal transduction in melanoma cells

**Figure 2:**
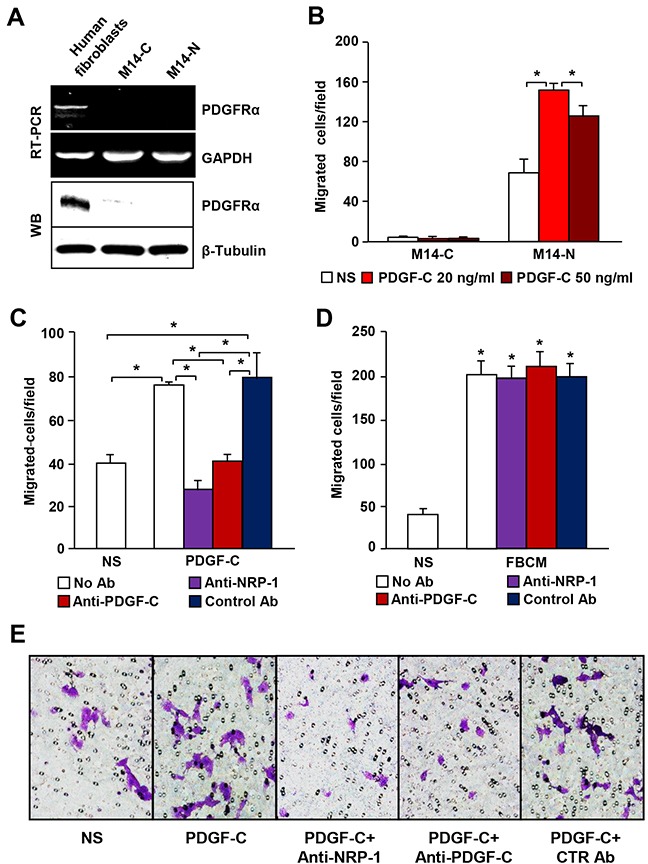
PDGF-C stimulates chemotaxis of PDGFRα negative M14-N cells through NRP-1 activation **(A)** PDGFRα expression in M14-N and M14-C cells was evaluated by RT-PCR and Western blot (WB) analyses. RNA or protein samples from human fibroblasts were included as positive control. GAPDH or β-tubulin detection was used as loading control for RT-PCR and WB analyses, respectively. **(B)** Migration of M14-C and M14-N cells in response to 20 or 50 ng/ml of PDGF-C was analyzed in Boyden chambers equipped with gelatin-coated filters and including the growth factor in the lower compartment. Data are expressed in terms of number of migrating cells per microscopic field. Each value represents the mean of three independent experiments (± SD). ANOVA followed by Bonferroni’s post-hoc test: p<0.05 (*). NS, non-stimulated cells. **(C)** The effect of blocking antibodies specific for NRP-1 or PDGF-C or IgG control antibody on the chemotactic response of M14-N cells to PDGF-C (20 ng/ml) was analysed in Boyden chambers equipped with gelatin-coated filters and including the growth factor in the lower compartment. Each value represents the mean of three independent experiments (± SD). ANOVA followed by Bonferroni’s post-hoc test: p<0.05 (*). NS, non-stimulated cells. **(D)** The specificity of M14-N cell chemotactic response to PDGF-C was evaluated by analysing the effect of blocking antibodies (5 μg/ml) specific for NRP-1 or PDGF-C or IgG control antibody on chemotaxis induced by an unrelated stimulus (fibroblast conditioned media, FBCM), assayed as described in panel B. ANOVA followed by Bonferroni’s post-hoc test: unstimulated *vs* FBCM stimulated groups, p<0.05 (*). NS, non-stimulated cells. **(E)** Selected representative microscopic fields (x100 magnification) are shown that correspond to: non-stimulated cells (NS) and cells stimulated with 20 ng/ml PDGF-C in the absence of antibodies and in the presence of 5 μg/ml anti-NRP-1, anti-PDGF-C or goat IgG control antibodies (CTR Ab).

**Figure 3:**
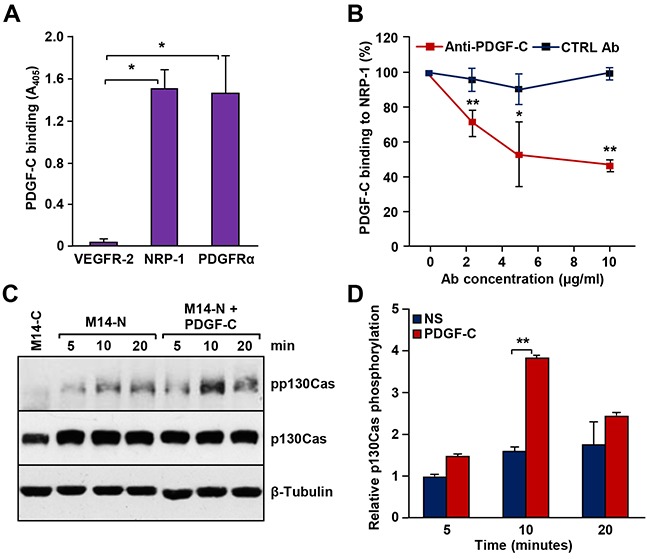
PDGF-C binds to NRP-1 *in vitro* and stimulates signal transduction in M14-N cells **(A)** PDGF-C binding to NRP-1 was assayed in Maxisorp Nunc immunoplates coated with the growth factor and incubated with a solution containing 1 μg/ml NRP-1/hFc chimera. Bound NRP-1/hFc chimeric polypeptide was quantified using an alkaline phosphatase-conjugated anti-human Fc antibody. PDGFRα/hFc and VEGFR-2/hFc chimeras were used as positive and negative controls, respectively. Each value represents the mean of three independent determinations (± SD). ANOVA followed by Bonferroni’s post-hoc test: p<0.05 (*). **(B)** The effect of pre-incubation of selected PDGF-C-coated wells with graded concentrations of goat anti-PDGF-C or IgG control antibodies on PDGF-C binding to NRP-1 was evaluated using the binding assay described in panel A. Student’s *t*-test: p<0.05 (*), p<0.01 (**). **(C)** M14-N cells were untreated or treated with 50 ng/ml PDGF-C for the indicated times and p130Cas phosphorylation levels were evaluated by Western blot. Total p130Cas was detected to determine the relative amount of phosphorylated protein and β-tubulin expression was tested as loading control. Protein extracts from M14-C cells, at time 5 min, were included in the analysis as negative control. A representative experiment out of three is shown. **(D)** Densitometric quantification of the levels of phospho-p130Cas relative to the total amount of p130Cas, after normalization by β-tubulin content in the samples. Histogram represents the mean values (± SD) of three independent determinations. Student’s *t*-test analysis: p<0.01 (**). NS, non-stimulated cells.

Since NRP-1 was reported to stimulate PDGFR signal transduction through interaction with PDGF-B [17], we hypothesized that PDGF-C might be involved, at least in part, in M14-N cell aggressiveness by co-activation of NRP-1 and PDGFRα. Thus, we initially investigated PDGFRα expression in M14-N cells, but the results of RT-PCR and Western blot analyses indicated that these cells do not express PDGFRα (Figure 2A). Nevertheless, M14-N cells migrated in response to PDGF-C stimulation (Figure 2B), and chemotaxis was specifically inhibited by PDGF-C and NRP-1 blocking antibodies, which instead did not affect M14-N cell migration in response to a nonspecific stimulus represented by fibroblast conditioned medium (Figure 2C–2E). These data, together with the high homology between VEGF-A and PDGF-C [15], suggest that PDGF-C directly binds NRP-1 leading to activation of NRP-1-dependent pathways. Indeed, using an *in vitro* cell-free binding assay on PDGF-C-coated 96-well plates, we demonstrated that PDGF-C was able to interact not only with PDGFRα, but also with NRP-1 (Figure 3A). Binding selectivity was indicated by the lack of PDGF-C interaction with VEGFR-2 (Figure 3A). Moreover, an anti-PDGF-C antibody specifically inhibited PDGF-C/NRP-1 interaction in a concentration-dependent manner, with an IC_50_ of ∼5 μg/ml (Figure 3B).

In other cellular systems, the p130Cas adaptor protein has been shown to play a crucial role in cell migration promoted by activation of NRP-1 after forming a co-receptor complex with PDGFRα or other tyrosine kinase receptors [17, 18]. Thus, we investigated whether activation of NRP-1 by PDGF-C, in the absence of PDGFRα, can also trigger p130Cas activation. Interestingly, exposure of M14-N cells to PDGF-C resulted in a time-dependent increase of p130Cas phosphorylation (Figure 3C and 3D), suggesting that PDGF-C interaction with NRP-1 triggers a downstream signal transduction.

### PDGF-C stimulates ECM invasion and vasculogenic mimicry of NRP-1 positive melanoma cells

**Figure 4:**
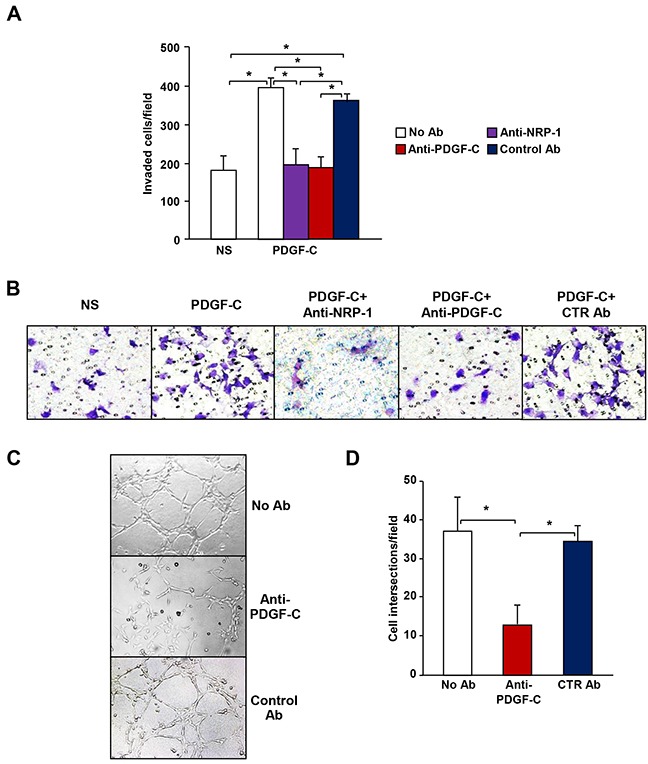
PDGF-C promotes the aggressive phenotype of M14-N cells **(A)** The effect of anti-PDGF-C and anti-NRP-1 blocking antibodies or IgG control antibody on ECM invasion by M14-N cells in response to PDGF-C (20 ng/ml) was analysed using Boyden chambers with matrigel-coated filters and including the growth factor in the lower compartment. Each value represents the mean of three independent determinations (± SD). ANOVA analysis followed by Bonferroni’s post-hoc test: p<0.05 (*). NS, non-stimulated cells. **(B)** Selected representative microscopic fields (x100 magnification) are shown that correspond to: non-stimulated cells (NS) and cells stimulated with 20 ng/ml PDGF-C in the absence of antibodies, in the presence of 5 μg/ml anti-PDGF-C and anti-NRP-1 or IgG control antibody (CTR Ab). **(C)** Formation of tube-like structures on matrigel was evaluated in untreated M14-N cells and in cells treated with 5 μg/ml of anti-PDGF-C or goat IgG control antibodies (CTR Ab). Photographs were taken after 6 h incubation (x100 magnification). **(D)** Histogram represents the number of cell intersections, counted in ten different microscopic fields for each experimental group. Each value represents the mean of three independent determinations (± SD). ANOVA analysis followed by Bonferroni’s post-hoc test: p<0.05 (*).

NRP-1 expression has been correlated with melanoma metastatic potential [5, 6, 9, 19, 20] and our findings on its activation by PDGF-C suggest that a PDGF-C/NRP-1 autocrine loop might sustain, at least in part, the aggressive phenotype of NRP-1 positive melanoma cells. In fact, we found that PDGF-C stimulated ECM invasion by M14-N cells was specifically abrogated by PDGF-C and NRP-1 blocking antibodies (Figure 4A and 4B).

Another characteristic of highly aggressive melanoma cells is their ability to form tube-like structures similar to vascular vessels formed by endothelial cells (vasculogenic mimicry), which *in vivo* favour nutrient supply for tumour growth and metastatic spreading. *In vitro* vasculogenic mimicry is measured by inducing the formation of tube-like structures in cells grown on a matrigel layer. As previously shown, M14-N cells spontaneously form tube-like structures (6), and here we found that PDGF-C specific antibodies markedly impaired this M14-N property (Figure 4C and 4D). Interestingly, VEGF-A neutralizing antibodies did not significantly reduce the formation of tube-like structures by M14-N cells (Supplementary Figure 2).

### The epithelial-mesenchymal transition (EMT) related genes zinc-finger E-box-binding 1 (ZEB1) and Snail are highly expressed in NRP-1/PDGF-C positive melanoma cells

**Figure 5:**
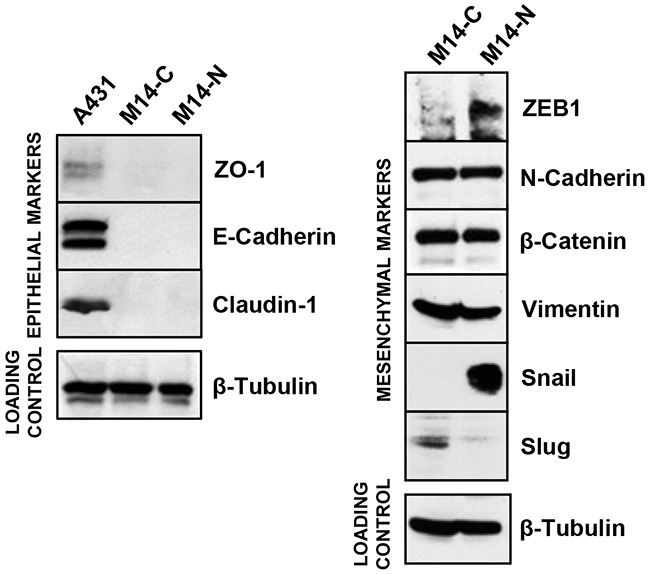
Expression of EMT markers in M14 cell clones Protein extracts from M14-C and M14-N cells were analysed for the expression of EMT markers by Western blot, using an EMT antibody sampler kit from Cell Signalling. Protein extracts from the human epidermoid carcinoma A431 cell line was used as positive control for the analysis of ZO-1, E-Cadherin and Claudin expression. Expression of β-tubulin was determined as a loading control. The results are representative of two different experiments.

EMT is a process that consists in the trans-differentiation of epithelial cells into cells with a mesenchymal phenotype, endowed with increased adhesive, invasive, and migratory properties. This process represents a switch in cell differentiation and behaviour that contributes to cancer progression, and is mediated by key transcription factors, including Snail, Slug and ZEB1 [21, 22]. Phenotype-switching between differentiated and invasive melanoma states is thought to be driven by oncogenic signalling and microenvironment influence, and it is coupled with a signalling switch of EMT-inducing factors [23]. To investigate the phenotype of M14-C and M14-N cell clones, we evaluated the expression of a set of epithelial (Zona occludens protein ZO-1, E-cadherin, Claudin-1) and mesenchymal (ZEB1, N-cadherin, β-catenin, Vimentin, Snail, Slug) markers by Western blot analysis. Results showed that even though both cell clones expressed N-cadherin, β-catenin and Vimentin and lacked ZO-1, E-cadherin and Claudin-1, only the more aggressive M14-N cell clone presented high levels of ZEB1 and Snail (Figure 5), suggesting an important role for these two molecules in its invasive characteristics. Interestingly, Slug, a member of the Snail family of transcription factors known to contribute to EMT by reducing cell adhesion through inhibition of integrin expression [24], was found to be down-modulated in M14-N cells (Figure 5). This finding is consistent with the increased adhesive properties to several ECM components and elevated integrin expression displayed by M14-N cells as compared to the less aggressive M14-C cells [6].

### PDGF-C silencing in M14-N cells reduces their invasive behaviour, vasculogenic mimicry and Snail expression

**Figure 6:**
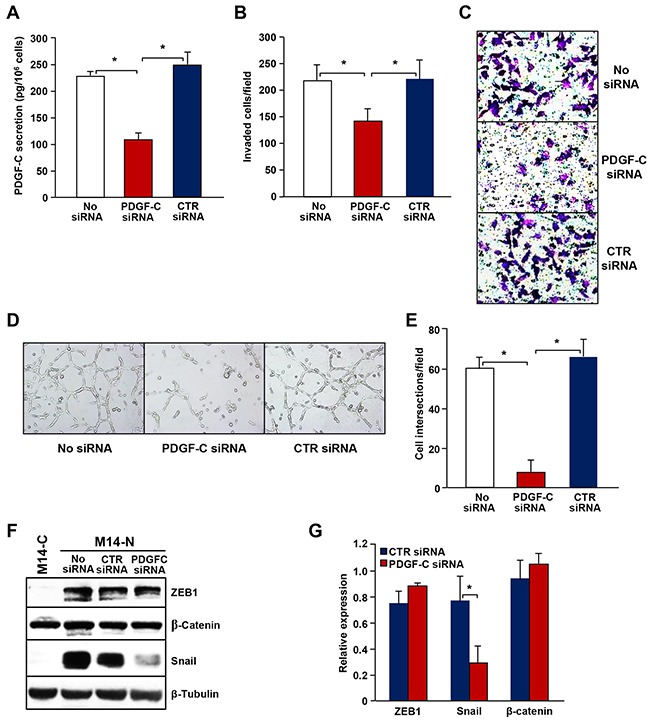
PDGF-C silencing in M14-N cells inhibits ECM invasion, vasculogenic mimicry and Snail expression **(A)** PDGF-C secretion by M14-N cells non-transfected (No siRNA) or transfected with PDGF-C specific or control siRNAs, quantified by ELISA. Each value represents the mean of four independent determinations (± SD). ANOVA analysis followed by Bonferroni’s post-hoc test: p<0.05 (*). **(B)** The effect of PDGF-C silencing by transfection of a specific siRNA on spontaneous ECM invasion by M14-N cells was evaluated using Boyden chambers equipped with matrigel-coated filters and containing migration medium in the lower compartment. Histogram represents the quantification of invaded cells and each value represents the mean of four independent determinations (± SD). ANOVA analysis followed by Bonferroni’s post-hoc test: p<0.05 (*). **(C)** Selected representative microscopic fields (x100 magnification) are shown that correspond to: M14-N cells non-transfected (No siRNA) and transfected with PDGF-C specific or control siRNAs. **(D)** Formation of tube-like structures on matrigel by M14-N cells non-transfected (No siRNA) or transfected with PDGF-C specific or control siRNAs. Photographs were taken after 6 h of cell incubation on matrigel (x100 magnification). **(E)** Histogram represents the number of cell intersections, counted in ten different microscopic fields for each experimental group. Each value represents the mean of three independent determinations (± SD). ANOVA analysis followed by Bonferroni’s post-hoc test: p<0.05 (*). **(F)** Extracts from M14-N cells, non-transfected (No siRNA) or transfected with control or PDGF-C specific siRNAs, and from M14-C cells as control, were analysed by Western blot for the expression of ZEB1, β-catenin or Snail as described in the legend of Figure 5. Expression of β-tubulin was determined as loading control. Photographs show the results of a representative experiment out of three. **(G)** Densitometric analysis of ZEB1, Snail or β-catenin immunoreactive bands normalized for the expression of β-tubulin. Bars represent the mean (± SD) of the results obtained from three independent experiments. Student’s *t*-test: p<0.05 (*).

The relevance of PDGF-C production in M14-N cell aggressive properties was confirmed by gene silencing experiments. In fact, transfection of a specific PDGF-C siRNA, which resulted in 60% inhibition of growth factor secretion (Figure 6A), significantly reduced M14-N cells ability to spontaneously invade ECM (Figure 6B and 6C) and almost completely inhibited tube-like structures formation (Figure 6D and 6E). We also analysed the influence of PDGF-C silencing on the expression of the EMT-related transcription factors ZEB1 and Snail in M14-N cells. At the time of analysis (i.e., 72 h after transfection) Snail expression was drastically reduced, whereas ZEB1 was not significantly modulated (Figure 6F and 6G). Conversely, β-catenin expression, which is equally expressed in the NRP-1-positive and NRP-1-negative cell clones, was not affected by PDGF-C silencing. These data suggest a role for Snail in the pathway triggered by PDGF-C/NRP-1 autocrine loop that sustains the aggressive phenotype of M14-N cells.

### PDGF-C/NRP-1 autocrine loop in other melanoma cells

**Figure 7:**
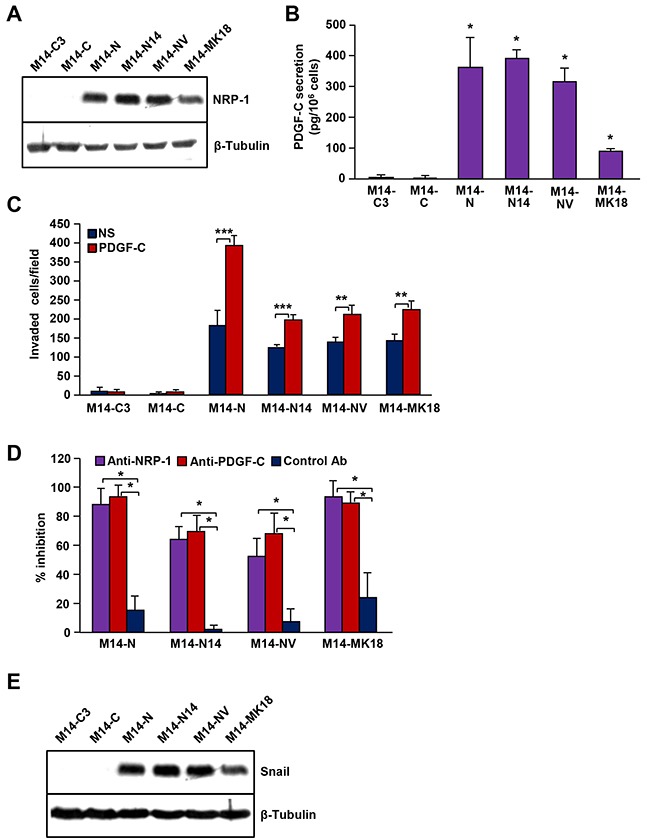
PDGF-C/NRP-1 autocrine loop in different human melanoma cell clones derived from M14 cells **(A)** Protein extracts from a set of M14-derived cell clones were analysed for NRP-1 expression by Western blot. Expression of β-tubulin was determined as a loading control. The results are representative of one out of two independent determinations. **(B)** PDGF-C secretion by M14 cell clones was measured in cell culture supernatants by ELISA. Data are the mean (± SD) of three independent determinations. ANOVA analysis followed by Bonferroni’s post-hoc test: p<0.05 (*). **(C)** The effect of PDGF-C on ECM invasion by M14-N cells was analysed using Boyden chambers with matrigel-coated filters and including the growth factor in the lower compartment. Histogram represents the number of invaded cells per microscopic field and data are the mean (± SD) of three independent determinations. Student’s *t*-test: p<0.01 (**); p<0.001 (***). NS, non-stimulated cells; PDGF-C, cells stimulated with 20 ng/ml PDGF-C. **(D)** The effect of anti-NRP-1 and anti-PDGF-C blocking antibodies or IgG control antibody (5 μg/ml) on ECM invasion by M14-N cells in response to PDGF-C (20 ng/ml) was analysed using Boyden chambers as described in C. Data are the mean percentage inhibition values (± SD) of ECM invasion in the presence of blocking antibodies, calculated from three independent determinations. ANOVA analysis followed by Bonferroni’s post-hoc test: p<0.05 (*). **(E)** Protein extracts from the indicated M14-derived cell clones were analysed for Snail expression by Western blot. Expression of β-tubulin was determined as a loading control. The results are representative of one out of two independent determinations.

To exclude the possibility that the PDGF-C/NRP-1 effects observed in M14-N cells were the consequence of clonal variation, we analysed other M14-derived melanoma clones: M14-N14 (NRP-1 positive and VEGFR-2 negative); M14C2/MK18 and M14-NV (NRP-1 and VEGFR-2 positive); M14-C3 and M14-C (NRP-1 and VEGFR-2 negative) [5, 6, 9]. Only cell clones expressing NRP-1 (Figure 7A) and secreting PDGF-C (Figure 7B) highly invaded ECM (Figure 7C) and their invasive ability was further stimulated by exposure to exogenous PDGF-C (Figure 7C). ECM invasion in response to PDGF-C was similarly down-regulated by NRP-1 and PDGF-C neutralizing antibodies (Figure 7D). Interestingly, Snail protein was detected exclusively in NRP-1 proficient cells (Figure 7E).

**Figure 8:**
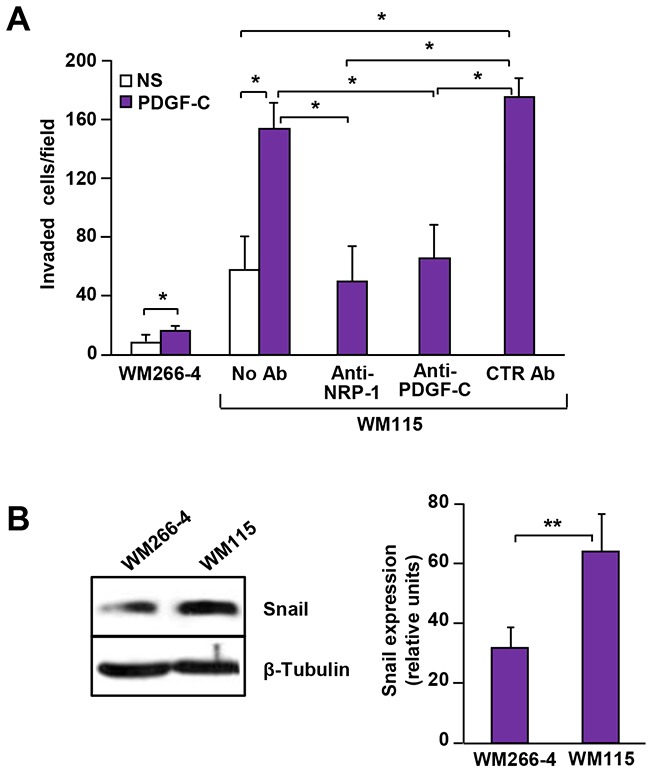
PDGF-C/NRP-1 autocrine loop in human melanoma cell lines derived from the primary lesion and from a lymph-node metastasis of the same patient **(A)** The effect of PDGF-C stimulation on ECM invasion by WM115 and WM266-4 cells was analysed using Boyden chambers with matrigel-coated filters, in the absence of antibodies or in the presence of 5 μg/ml anti-NRP-1, anti-PDGF-C or IgG control antibodies, including the growth factor in the lower compartment. Histogram represents the number of invaded cells per microscopic field and data are the mean of three independent determinations (± SD). NS, non-stimulated cells; PDGF-C, cells stimulated with 20 ng/ml PDGF-C. ANOVA analysis followed by Bonferroni’s post-test: p<0.05 (*). **(B)** Protein extracts from WM115 and WM266-4 cells were analysed for Snail expression by Western blot. Expression of β-tubulin was determined as a loading control. Histogram represents the relative levels of Snail expression, calculated by densitometric analysis and normalized by β-tubulin expression in each sample. Values are the mean (± SD) of the results from three independent determinations. Student’s *t*-test: p<0.01 (**).

PDGF-C/NRP-1 autocrine loop activity was also explored in two human melanoma cell lines derived from the same patient: one originated from the primary tumour (WM115) and the other from a lymph-node metastasis (WM266-4). We previously demonstrated that NRP-1 plays a relevant role in the ability of WM115 cells to invade ECM [6] and that these cells also express high PDGF-C levels [9]. By contrast, WM266-4 cells, which are much less efficient in invading ECM, show low NRP-1 expression and do not produce PDGF-C [6, 9]. Accordingly, we found that PDGF-C strongly induced ECM invasion by WM115 cells, while it only slightly stimulated WM266-4 invasiveness. Moreover, NRP-1 and PDGF-C neutralizing antibodies similarly inhibited the invasive response of WM115 cells to PDGF-C (Figure 8A). Snail expression was also higher in WM115 cells compared to WM266-4 cells (Figure 8B).

Overall, these results strengthen the hypothesis that the PDGF-C/NRP-1 autocrine loop may contribute to the maintenance of an aggressive phenotype in melanoma cells.

## DISCUSSION

In the present study, we demonstrate for the first time that PDGF-C, in the absence of PDFGRα, is able to bind NRP-1 and that this interaction contributes to the aggressive phenotype of melanoma cells by activation of specific signal transduction pathways.

PDGF-C is a pleiotropic growth factor with important roles not only in regulation of neural and vascular functions but also in tumour development and growth [10, 12]. In fact, it is abundantly produced by a plethora of cells present in the tumour microenvironment (i.e., endothelial cells, vascular smooth muscle cells, pericytes, vascular fibroblasts, tumour associated macrophages) [10] and can be also secreted by tumour cells [9, 14, 25].

We previously reported that PDGF-C transcript is widely expressed in melanoma, being detected in 80% (4/5) and 87% (13/15) of the primary and metastatic cell lines tested, respectively [9]. By contrast, PDGF-C mRNA is present at very low levels in only 17% (1/6) of melanocyte samples. Moreover, *in vitro* and *in vivo* studies performed by our group and other authors have demonstrated that NRP-1 is involved in melanoma invasiveness [5, 6, 9, 16, 20, 26]. In fact, activation of this membrane receptor results in the acquisition of several properties that characterize the metastatic phenotype of melanoma cells [5, 6, 16]. The high PDGF-C homology to VEGF-A [15] prompted us to investigate whether this growth factor may also interact with the VEGF-A co-receptor NRP-1 and co-operate with it in promoting melanoma aggressiveness.

This study provides evidence that in human melanoma cells expressing NRP-1 but devoid of other VEGFRs and PDGFRα expression, an autocrine PDGF-C/NRP-1 loop sustains several characteristics of melanoma aggressive phenotype. In fact, these cells secrete high PDGF-C levels in culture media and PDGF-C neutralizing antibodies or PDGF-C silencing impair melanoma invasiveness and vasculogenic mimicry. Moreover, NRP-1 expressing cells specifically migrate in response to exogenous PDGF-C and NRP-1 blocking antibodies abrogate PDGF-C induced chemotaxis. Consistently, NRP-1 negative melanoma cells fail to migrate toward the growth factor. These data, together with the finding that PDGF-C directly interacts with NRP-1 *in vitro*, support the notion that NRP-1 expression is sufficient for PDGF-C to activate signalling pathways involved in melanoma invasiveness. Indeed, melanoma cell exposure to PDGF-C results in increased p130Cas phosphorylation, which is a downstream effector of NRP-1 [18].

In order to understand the molecular mechanisms involved in the melanoma invasive phenotype induced by PDGF-C, we have investigated the pattern of EMT-related protein expression in NRP-1-negative and -positive cells. In fact, the acquisition of a mesenchymal phenotype by epithelial cells is thought to confer many solid tumours higher migratory and invasive properties by disrupting cell to cell junctions [27]. In melanoma, a number of molecules involved in EMT have been implicated in the promotion of a metastatic phenotype [28, 29]. NRP-1 and PDGF-C positive M14-N cells, which are highly invasive and possess a strong ability to form tube-like structures similar to blood vessels [6], display a pattern of adhesion/migration markers corresponding to the mesenchymal phenotype. In particular, they show high expression of ZEB1 and Snail, two EMT-inducing transcription factors crucial for the acquisition of an invasive behaviour by melanoma cells [30, 31]. Accordingly, NRP-1 and PDGF-C negative M14-C cells, which have low invasive potential and lack vasculogenic mimicry [6, 9], do not express these transcription factors.

Conversely, Slug, a member of the Snail family of transcriptional repressors that is detected in the M14-C clone, results to be down-modulated in M14-N cells. Slug is strongly expressed in neural crest-delaminated melanoblasts, melanocytes and benign nevi [32] and, together with another transcription factor of the zinc-finger E-box-binding like family (ZEB2), is regarded as a positive prognostic factor for melanoma patients [30, 31]. ZEB1 co-expression with TWIST, a basic helix-loop-helix transcription factor whose activation plays an essential role in EMT and cancer metastasis, is instead considered an indicator of tumour aggressiveness. Actually, a switch from ZEB2/Slug (differentiated state) to ZEB1/TWIST (oncogenic invasive phenotype) expression indicates malignant progression. Moreover, Slug allows the reversion of ZEB1/TWIST-driven invasive phenotype toward a differentiated phenotype [30]. Therefore, our findings on Slug and ZEB1 expression pattern in M14 cell clones are consistent with the more aggressive phenotype of M14-N compared to M14-C cells.

Slug and Snail have opposite effects on tumour cell migration, adhesion and integrin expression. Snail promotes mesenchymal cell motility by Rac1 activation, whereas Slug inhibits cell migration through Rho signalling [33]. Moreover, differently from Slug that represses integrin expression [24], Snail increases cell adhesion and motility by enhancing the expression of the collagen binding integrin α2β1 [33], which is highly expressed in M14-N cells [6]. Interestingly, Snail expression in M14-N cells is down-modulated by PDGF-C silencing, suggesting a role for this molecule in the aggressive phenotype promoted by PDGF-C. Snail has also been implicated in various processes related to cell differentiation and survival and many signalling molecules may induce Snail expression in response to stimuli produced in tumour microenvironment [34, 35]. Actually, Snail is detected in several types of cancer and its expression correlates with increased migration, invasion, and metastasis. Moreover, advanced disease stages and poor prognosis are frequently accompanied with high Snail levels in the tumour [36]. In particular, Snail over-expression in melanoma correlates with low levels of E-cadherin and confers tumour cells both invasive and immunosuppressive properties [37], whereas Snail mRNA is not detected in primary melanocytes [38]. Therefore, based on our findings in different M14-derived clones and other melanoma cell lines expressing NRP-1 and PDGF-C, it can be hypothesized that PDGF-C requires Snail to stimulate ECM invasion and vasculogenic mimicry of melanoma cells.

In conclusion, evidences are presented demonstrating NRP-1 activation by PDGF-C and strongly suggesting that PDGF-C/NRP-1 signaling might be relevant in the metastatic switch of melanoma cells, even in the absence of PDGFRα. In NRP-1 proficient melanomas, microenvironmentally- or tumour-secreted PDGF-C may activate specific signal transduction pathways (including p130Cas phosphorylation) and transcription factors (like Snail), which promote an invasive phenotype.

## MATERIALS AND METHODS

### Reagents and cell lines

Cell culture media and reagents were purchased from Lonza (Basel, Switzerland); foetal bovine serum (FBS) was from Euroclone (Pero, Italy) and fatty acid-free bovine serum albumin (BSA) from Roche (Mannheim, Germany). Anti-VEGF-A (AF293) and anti-PDGF-C (AF1560) neutralizing polyclonal antibodies, ELISA assay for recombinant human PDGF-C quantification, NRP-1/hFc, VEGFR-2/hFc and PDGFRα/hFc chimeric proteins and the normal goat IgG control antibody used as negative control in the functional assays were from R&D Systems (Abingdon, UK). The rabbit polyclonal anti-human NRP-1 antibody H286 was from Santa Cruz (Santa Cruz, CA). rhPDGF-C was from ReliaTech GmbH (Wolfenbuttel, Germany).

The origin and culture conditions of the M14-derived clones were previously described [5, 6, 9]. M14-N and M14-N14 clones are highly invasive cell lines expressing NRP-1 but not VEGFR-2 [5, 6]; M14-NV and M14C2/MK18 clones are also highly aggressive and are positive for both NRP-1 and VEGFR-2 [5, 9]; M14-C and M14-C3 clones are devoid of VEGFR-2 and NRP-1 expression [5, 6, 9].

Normal human fibroblasts were kindly provided by the Laboratory of Molecular and Cell Biology, IDI-IRCCS, Rome, Italy, and the human epidermoid carcinoma A431 cell line, as well as the human melanoma cell lines WM115 and WM266-4 were obtained from ATCC (Manassas, VA).

### Evaluation of PDGF-C secretion

Semi-confluent melanoma cell cultures were incubated in 0.1% BSA/RPMI 1640 medium without FBS for 24 h. Culture supernatants were, then, collected, centrifuged at 600xg for 10 min to remove cells in suspension, concentrated at least ten-fold in Centriplus concentrators (Amicon, Beverly, MA) and frozen at −80°C till use. Cells were detached from the flasks with a solution of 1 mM EDTA in PBS and the total cell number/culture was recorded. Quantification of the amount of PDGF-C in the concentrated supernatants was performed using a human Quantikine immunoassay kit (R&D Systems), according to the manufacturer’s directions.

### Chemotaxis and ECM cell invasion assays

*In vitro* invasion assay was performed using Boyden chambers equipped with 8-μm pore diameter polycarbonate filters (Nuclepore, Whatman Incorporated, Clifton, NJ), coated with 20 μg of matrigel (BD Biosciences, Buccinasco, Italy) [5]. Briefly, melanoma cells were collected from continuous cultures, washed, suspended in migration medium (1 μg/ml heparin/0.1% BSA in RPMI 1640), and loaded (1×10^5^ cells) into the upper compartment of the Boyden chambers. Migration medium, alone or containing the indicated stimulus, was added to the lower compartment of the chambers. For each experimental condition, three Boyden chambers were set up. After incubation of the Boyden chambers at 37°C in a CO_2_ incubator for 3 h, filters were removed, fixed in ethanol for 5 min and stained in 0.5% crystal violet for 15 min. Non-migrating cells were removed from the upper surface of the filter by wiping with a cotton swab and migrated cells, attached to the lower surface of the filter, were counted under the microscope. Twelve high-magnification microscopic fields (x40 magnification), randomly selected on triplicate filters, were scored for each experimental condition. Where indicated, invasion assays were performed in the presence of antibodies directed against selected molecules, after cell pre-incubation with these antibodies for 30 min at room temperature, in a rotating wheel.

The chemotactic response of melanoma cells to different stimuli was analysed as described for invasion assay, using polycarbonate filters coated with 5 μg/ml gelatin solution and allowing cells (1.5×10^5^) to migrate for 18 h, as previously reported [39].

Conditioned medium from normal human fibroblasts, M14-C or M14-N cells, used in selected experiments as chemotactic stimulus, was produced as described above and included in the lower compartment of the Boyden chambers.

### RT-PCR analysis

cDNA preparation and PCR amplification were performed as previously described [40], utilizing an annealing temperature of 58°C and the following primers: human PDGFRα, forward primer 5’-GATTGTGGTCACCTGTGCTG-3’, reverse primer 5’-GGCGTGCCTTCAGCTGTGCA-3’ (633 bp fragment); GAPDH, forward primer 5’-TCCCATCACCATCTTCCA-3’, reverse primer 5’-CATCACGCCACAGTTTCC-3’ (380 bp fragment).

### Western blot analysis

SDS-PAGE electrophoresis and transfer of separated polypeptides to nitrocellulose membranes (GE Healthcare Europe, Milano, Italy) was performed by standard techniques using 60 μg of proteins per sample in 7-12% gradient SDS-polyacrylamide gels. Detection of the different polypeptides under study was performed using the following primary antibodies: anti-PDGFRα (polyclonal rabbit antibody C20) and anti-NRP-1 (monoclonal mouse antibody A12) from Santa Cruz Biotechnology (Santa Cruz, CA); anti-p130Cas (monoclonal rabbit antibody E1L9H) and anti-phospho-p130Cas (rabbit polyclonal antibody Tyr410) from Cell Signaling Technology (Danvers, MA); anti-β-tubulin rabbit polyclonal antibody H-235 from Santa Cruz Biotechnology; EMT markers, from the Cell Signaling EMT antibody sampler kit.

### PDGF-C binding assays

Quantification of PDGF-C binding to NRP-1 was performed on 96-well Maxisorp Nunc immunoplates (Nunc, Roskilde, Denmark) coated with 1 μg/ml PDGF-C in PBS. After blocking of the plates with 2% BSA (w/v) in PBS (hereafter referred to as blocking buffer), 50 μl of NRP-1/hFc chimera (1 μg/ml) were added to selected wells and plates incubated at 37°C for 30 min in blocking buffer supplemented with 1 mM CaCl_2_ and MgCl_2_. The amount of chimera bound to the cytokine was quantified by incubating wells with an anti-human Fc alkaline phosphatase-conjugated goat IgG (1:2.000) at room temperature for 1 h. After alkaline phosphatase reaction, optical density at 405 nm was measured in a Microplate reader 3550-UV (Bio-Rad, Hercules, CA). The specificity of PDGF-C binding to NRP-1 was investigated by pre-incubating at room temperature for 30 min selected PDGF-C-coated wells with graded concentrations (2.5-10 μg/ml) of a neutralizing antibody (goat anti-human PDGF-C, from R&D Systems) or control normal goat IgG (R&D Systems), before adding the NRP-1/hFc chimera. Background was determined in blocking buffer-coated wells. As positive and negative controls of PDGF-C binding, selected wells were incubated with (1 μg/ml) PDGFRα/hFc or VEGFR-2/hFc chimeras, respectively.

### Differentiation of melanoma cells in tube-like structures

For the analysis of tube-like structures on matrigel, the matrix was allowed to thaw overnight at 4°C and diluted 1:3 in serum-free RPMI 1640 medium. Diluted matrigel (100 μl) was layered onto 24-well plates that were then incubated at 37°C for 30 min, to allow matrigel solidification, as described [41]. Melanoma cells were suspended in complete culture medium (1×10^5^ cells in 0.5 ml), dispensed onto the solidified matrix and incubated at 37°C in a 5% CO_2_ environment. At the indicated times, plates were photographed using a Leica inverted microscope and a Canon digital camera PowerShot G5. The formation of tube-like structures was quantified by counting the number of cell intersections in ten different microscopic fields per group (x100 magnification).

### PDGF-C gene knockdown

M14-N cells were transfected with either siRNA targeting the PDGF-C gene (ID s31862, Life Technologies, Carlsbad, CA) or non-targeting siRNA (Silencer Negative Control #1 siRNA, Life Technologies) at a final concentration of 20 nM, using lipofectamine RNAiMAX in OptiMEM serum-free medium (Life Technologies), according to the manufacturer’s instructions. Cells were harvested 72 h after transfection and subjected to further analyses.

### Statistics

Results were expressed as arithmetic means ± standard deviation (SD) and significance of the differences was tested by unpaired, two-tailed Student’s *t*-test, when two means were compared, or ANOVA followed by Bonferroni’s post-hoc test, for multiple comparison; p values <0.05 (*), <0.01 (**) and <0.001 (***) for Student’s *t*-test and <0.05 (*) for ANOVA analysis were considered significant.

## SUPPLEMENTARY MATERIALS FIGURES



## Abbreviations

BSA: bovine serum albumin
ECM: extracellular matrix
EMT: epithelial-mesenchymal transition
FBS: foetal bovine serum
MMPs: matrix metalloproteinases
NRP-1: neuropilin-1
PDGF: platelet-derived growth factor
PDGFR: PDGF receptor
VEGF: vascular endothelial growth factor
VEGFRs: VEGF receptors.
