# Supplementary material for: Platelet-derived growth factor-C promotes human melanoma aggressiveness through activation of neuropilin-1
Source: Oncotarget. 2017 Jun 27;8(40):66833–48. doi: 10.18632/oncotarget.18706 (PMC5620139; doi:10.18632/oncotarget.18706)
Supplement: Supplementary file 1 [file oncotarget-08-66833-s001.pdf]

Platelet-derived growth factor-C promotes human melanoma aggressiveness through activation of neuropilin-1

SUPPLEMENTARY MATERIALS

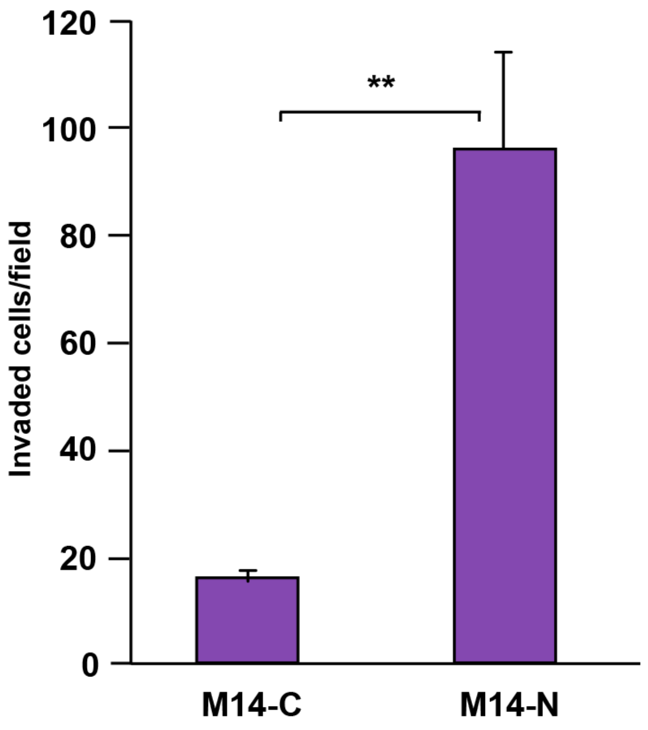

Supplementary Figure 1: M14-N conditioned medium does not stimulate ECM invasion by NRP-1 negative M14-C cells. M14-C cells ability to invade ECM in response to conditioned medium obtained from M14-N cells was analysed using Boyden chambers equipped with matrigel-coated filters and including the conditioned medium in the lower compartment. M14-N cells were used as positive control. Histogram represents the quantification of invaded cells per microscopic field. Each value is the mean number for three independent experiments (± SD). Student's *t*-test: $p<0.01$ (**).

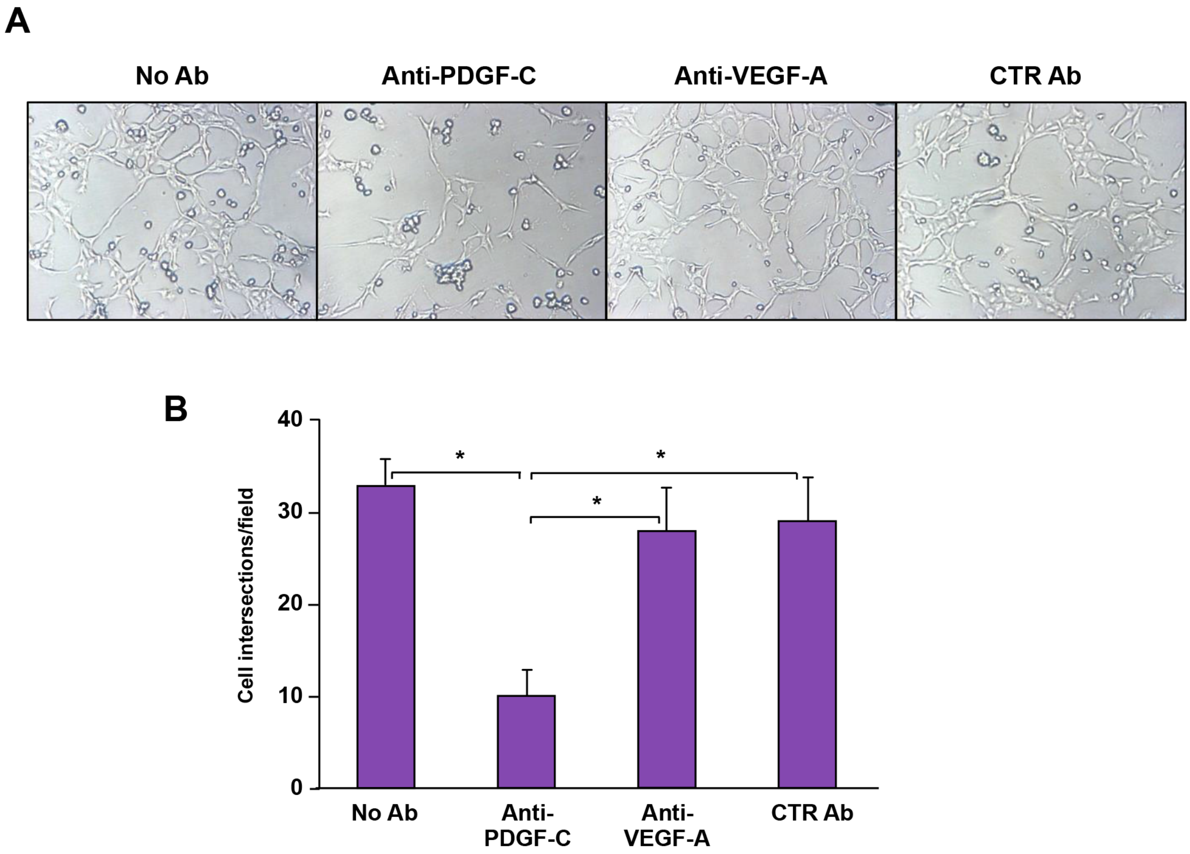

Supplementary Figure 2: VEGF-A neutralization does not significantly affect M14-N ability to form tube-like structures. (A) Formation of tube-like structures on matrigel was evaluated in M14-N cells, untreated (No Ab) or treated with 5 µg/ml of anti-PDGF-C, anti-VEGF-A or IgG control antibodies (CTR Ab). Photographs were taken after 24 h incubation (x100 magnification). **(B)** Histogram represents the mean number (± SD) of cell intersections from three independent experiments. ANOVA analysis followed by Bonferroni's post-hoc test: $p<0.05$ (*).
